# Cancer of the Tongue—Division of the Gustatory Nerve

**Published:** 1851-07

**Authors:** 


### ARTICLE XXV.

### *Cancer of the Tongue—Division of the Gustatory Nerve.*

Amongst the patients admitted under the care of Mr. Hilton, of Guy's Hospital, was a woman affected with malignant disease of the tongue, whose sufferings were so great, that an attempt was made to give her temporary relief by dividing the sentient nerve of the organ. Such direct surgical applications of physiological data afford an additional proof how advantageously anatomy, physiology and surgery, go hand in hand; and it is very gratifying to notice the anxiety Mr. Hilton evinced of doing something for his patient, in a case which baffled all attempts at permanent restoration. Division of nerves in physiological experiments is frequently performed; it has likewise been practiced, with but indifferent results, in tic doloureux; but we are not aware that the division of the gustatory was ever made before, with a view of blunting the sensation of the part, and, consequently, relieving pain.

Mr. Hilton's patient was a woman, about thirty-nine years of age, who was admitted in February, 1850, with a large, excavated, ragged ulcer, with hard, raised and everted edges, on the left side of the tongue, extending from the junction of the middle with the posterior third, to nearly the tip and the median line. The poor woman suffered much pain in the head and the left ear, which broke her rest; she was likewise troubled with a profuse flow of tenacious saliva, and unable to take solid food, as its passage over the ulcer gave exquisite pain. Mr. Hilton determined to remove the disease by excision; after, however, watching the effects of the actual cautery to the projecting and painful edges of the ulcer, and the administration of conium internally.

But intercurrent inflammation, and the formation of abscess in the submaxillary region, caused this line of practice to be altered, and Mr. Hilton, thinking that the above described symptoms might be relieved, if the sensibility of the ulcer could be destroyed, and knowing that the operation of sloughing off the diseased parts by the application of ligatures would thus be rendered nearly painless, determined to divide the lingual gustatory branch of the fifth nerve, between the diseased portion of the tongue and the brain.

On the 20th March, the patient was placed in the recumbent posture, but with the head a little raised, opposite a good light, and the tongue being drawn out and steadied by an assistant, Mr. Hilton divided vertically, with a small knife, the mucous membrane, and the sub-mucous tissue, for about three-quarters of an inch, over the hyoglossus muscle, and above the sublingual gland. The parts were, however, much obscured by the almost constant flow of venous blood into the wound, but by continuing the incision through the upper edge of the sublingual gland, the nerve was exposed. Mr. Hilton took hold of it with forceps, and divided it with scissors. All sensation was immediately lost in the anterior part of the tongue on the left side, as well as in the ulcer; and either part could be seized and pinched with the forceps, without the patient being aware of what was being done.

Some pains and inflammation in the soft parts of the face and neck, occurred soon after; these were relieved by appropriate means, but the profuse flow of saliva had ceased, and semi-liquid food could be taken into the mouth without occasioning uneasiness. Portions of the ulcer were subsequently separated by ligatures, the operation giving no pain; but it was found impossible to include the whole diseased mass in the threads.

One month after the division of the nerve, the patient regained slight feeling at the tip of the tongue on the left side, the flow of saliva recommenced, and the ulcer became somewhat painful to the touch. Her health being much weakened, a cervical gland enlarged, and the ulceration spreading, Mr. Hilton sent her back to the country, judging that the disease was too far advanced for any more active surgical interference. She returned to Yarmouth, where she died about four months after her discharge from the hospital.

By the division of the gustatory branch of the fifth nerve, the sensibility of the ulcer was destroyed for a time, the patient could take food without its passage giving any pain; the excessive flow of saliva was diminished, and the application of ligatures to the ulcer did not occasion any suffering. Mr. Hilton thinks that further experience is necessary to fix the line of practice in cases of this nature, but he considers that the division of the nerve should never be omitted when it is determined to remove the ulcerating mass by the application of the ligature, a process which is known to be extremely painful.

